# Predictive value of maternal serum β-hCG concentration in the ruptured tubal ectopic pregnancy

**Published:** 2015-02

**Authors:** Roya Faraji Darkhaneh, Maryam Asgharnia, Nastaran Farahmand Porkar, Ali Akbar Alipoor

**Affiliations:** 1 *Reproductive Health Research Center, Department of Obstetrics and Gynecology, Guilan University of Medical Sciences, Rasht, Iran.*; 2 *Guilan University of Medical Sciences, Rasht, Iran.*

**Keywords:** *Tubal ectopic pregnancy*, *Tubal rupture*, *Serum β-hCG concentration*

## Abstract

**Background:**

Measurement of serum β-hCG concentration commonly used to diagnose tubal ectopic pregnancy (EP) and follow up patients treated conservatively.

**Objective:**

The aim of this study was to determine the predictive value of maternal serum β-hCG concentration in ruptured tubal ectopic pregnancy to help physicians identify those women who are at greatest risk.

**Materials and Methods:**

This is a cross-sectional study conducted on all women with a diagnosis of tubal ectopic pregnancy who were treated in Alzahra Hospital, in Rasht, from March 2002 to February 2011. The data was collected for each woman from medical records and included age, parity, gravidia, gestational age, primary level of serum β-hCG, rupture status, past history of pelvic inflammation disease, EP, abortion, and intrauterine contraceptive device use. Women with tubal rupture were compared to those without rupture. Statistical analysis was conducted by SPSS 19 for Windows.

**Results:**

A total of 247 cases of tubal ectopic pregnancy were recorded during the study period. One hundred and ninety seven (79.8%) were cases with unruptured EP and 50 patients (20.2 %) were cases with ruptured EP. The mean level of β-hCG was significantly higher in patients with ruptured EP compared to patients with unruptured EP (p=0.03). Logistic regression analysis revealed that >1750 IU/ml of β-hCG levels (OR: 1.41; 95% CI: 1.18-1.68) was the significant risk factors for tubal rupture.

**Conclusion:**

Higher β-hCG levels seem to be significant risk factors for rupture of a tubal EP.

## Introduction

Ectopic pregnancy (EP) occurs when fertilized ovum implants outside endometrial cavity and the incidence is approximately 2% of all pregnancies (1, 2). The most common extra-uterine location is the fallopian tube, which accounts for 98% of all ectopic pregnancies (3). During last decades, the incidence of tubal ectopic pregnancy among women who go to an emergency department with first trimester bleeding, pain, or both has increased from 4.5 per 1000 pregnancies in 1970 to an estimated 19.7 per 1000 pregnancies in 1992 (4). 

Hemorrhage from rupture of tubal ectopic pregnancy is still the leading cause of pregnancy related maternal death in the first trimester and accounts for 10-15% of all pregnancy related deaths, despite improved diagnostic methods leading to earlier detection and treatment (1-5). The mortality ratio was 3.5 times higher for women older than 35 years than those younger than 25 years (5).

The knowledge of risk factors associated with the rupture of an EP may be a valuable tool to identify women who can be treated conservatively (1). Three factors specially may be considered as potential for tubal damage. The first factor, gestational age is proportional to the duration of exposure to the trophoblast-mediated erosive event on the tube. The second factor, ectopic mass diameter and free fluid in the pouch of Douglas as evaluated by transvaginal ultrasonography, is proportional to the extent of the tubal disruption. Finally, trophoblastic viability is indicative of the ability of the trophoblast to implant and infiltrate the tubal wall (6). 

β-hCG is secreted by syncytiotrophoblasts and the increase of serum hCG level indicates the presence of viable uterine pregnancies: β-hCG doubles every 1.5 days up to 5 weeks after the last menstrual period, and then every 3.5 days from the 7^th^ week (or when β-hCG is >10000 IU/L) (7). Although β-hCG concentrations do not increase at this rate in most ectopic pregnancies, measurement of β-hCG which is an accurate marker of trophoblastic viability is commonly used for diagnosis and follow-up in patients with EP (6-8). Some literature has found a positive correlation between serum β-hCG levels and tubal rupture. However, there is no adaptive on the level of β-hCG associated with likelihood of rupture (9). 

Thus in present study, we aimed to determine the predictive value of maternal serum β-hCG concentration for rupture in patient with tubal ectopic pregnancy.

## Materials and methods

This was a cross-sectional study conducted on all women with tubal ectopic pregnancy who were treated in the Al-Zahra Hospital in Rasht, after approving by the Ethical Committee of Guilan University of Medical Sciences, from March 2002 to February 2010. Patient with heteropic pregnancy was excluded from study. Ectopic pregnancy was confirmed by positive β-hCG and transvaginal ultrasonography findings in all patients. 

The ultrasonographic findings that designate the ectopic mass included gestational sac like anechoic mass surrounded by peripheral hyperechogenic halo that does not contain a viable embryo and located in the paraovarian region, solid or complex irregularly boarded mass which resembles a haematosalpinx or pelvic haematoma around the paraovarian region, and ectopic gestational. Moreover, gestational age was calculated according to the last menstrual period (LMP) at the time of admission. The information collected for all woman from medical records included; age, parity, gravidity, gestational age, serum β-hCG concentration at the time of admission in hospital, rupture status, past history of pelvic inflammatory disease (PID), previous EP, number of normal pregnancy, abortion history, and the use of intrauterine contraceptive device (IUD).

Ruptured EP for patients was defined according to physical examinations and ultrasonographic finding (including visualization of a ruptured extra uterine gestational sac and free fluid or haematoma in pelvic fossa). Finally, the study group was divided into two subgroups: ruptured EP and unruptured EP.

**Statistical analysis**

The results are expressed as mean±SD for continuous variables or percentage and number for categorical variables, as appropriate. Comparison of maternal, obstetric history and maternal serum β-hCG concentrations among two subgroups was performed by Student’s *t*-test and Mann-Whitney-Wilcoxon test for independent samples. Pearson’s chi-square and Fisher’s exact test were also applied for comparison of groups where appropriate. Receiver operating characteristic (ROC) curve analysis was used to find cut-off point for β-hCG variables. The sensitivity, specificity, positive predictive value (PPV) and negative predictive value (NPV) were calculated. Logistic regression analysis was used to identify predictors for tubal rupture in EP. The Statistical Package for the Social Sciences, version 18.0, SPSS Inc, Chicago, Illinois, USA were used for the data analyses. P<0.05 was considered to indicate statistical significance.

## Results

A total of 249 cases of tubal ectopic pregnancy were recorded during the study period. Two patients were excluded for heteropic pregnancy. Of these, 197 patients (79.8%) were cases with unruptured and 50 (20.2%) were cases with ruptured tubal ectopic pregnancies. Age was a statistically significant factor between women with unruptured and ruptured tubal ectopic pregnancies (p<0.0001). The mean gestational age was significantly lower and mean level of β-hCG was significantly higher in the ruptured group compared with the unruptured group (p=0.01 and p=0.03, respectively). 

**Figure 1:**
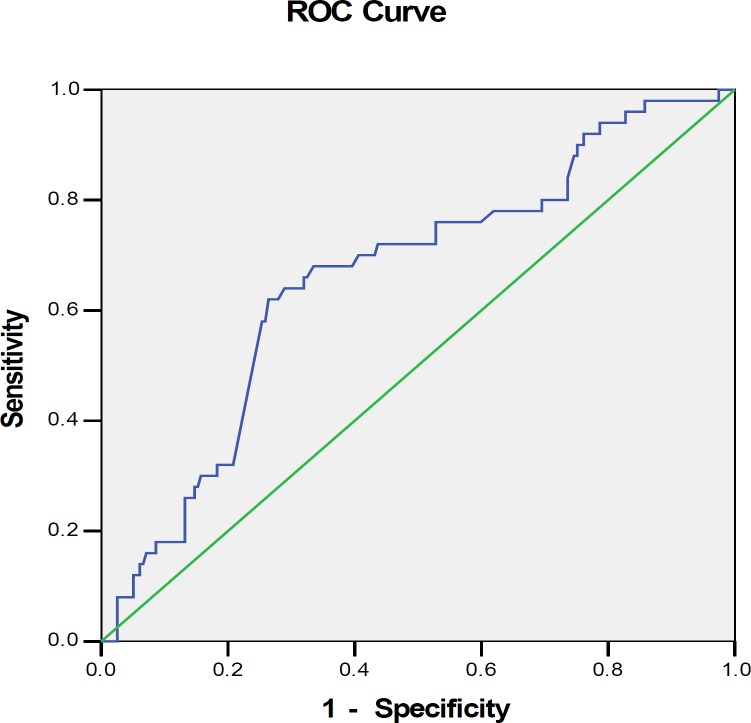
ROC curves for a prediction of tubal rupture using serum β-hCG. Receiver operating characteristic (ROC) curves showing true-positive rate (sensitivity) and false-positive rate (1- specificity) for each of several potential beta-human chorionic gonadotropin (β-hCG) cut-points to predict ruptured tubal ectopic pregnancy (β-hCG ≥cutoff).

Ectopic pregnancy was occurred in the right tubal in 109 patients (55.33%) of unruptured groups and in left tubal in 27 patients (60%) of ruptured groups. No significant associations existed regarding parity, gravidity, the number of previous normal pregnancies, past history of PID, previous EP, abortion history, and IUD use (Table I). The ROC curve for a prediction of tubal rupture using serum β-hCG is shown in figure 1. The serum β-hCG concentration for the best prediction of tubal rupture was 1750 IU/mL, with cut-off value showing a sensitivity of 62%, a specificity of 73.6%, a PPV of 37.3% and a NPV of 88.4%. univariate analysis revealed that >1750 IU/ml of β-hCG levels (OR: 1.41; 95% CI: 1.18-1.68) was the significant risk factors for tubal rupture. Multivariate logistic regression analysis identified that tubal rupture occurred more often in patients who are older than 35 (p=.009) and in women whose β-hCG level is upper than 1750 IU/ml (p<0.0001) (Table II).

**Table I:** Details of women which unruptured and ruptured tubal ectopic pregnancy

		**Unruptured group (n= 197) n (%)**	**Ruptured group (n=50) n (%)**	**p-value**
Age (yr)				
	Mean ± SD ( range)	28.69 ± 0.7 (16-45)	32.34 ± 5.14 (20-40)	<0.0001
	< 20	10 (5.08 %)	1 (2.00%)	<0.001
	21- 35	161 (81.73 %)	33 (66.00 %)	
	> 36	26 (13.20%)	16 (32.00 %)	
Parity				
	1	107 (54.31 %)	23 (46.00 %)	0.26
	2	52 (26.40%)	11 (22.00%)	
	3	28 (14.2 1%)	11 (22.00 %)	
	4	10 (5.08%)	5 (10.00%)	
Gravidity				
	1	78 (39.60%)	16 (32 %)	0.44
	2	58 (29.44%)	14 (28 %)	
	≥ 3	61 (30.96%)	20 (40%)	
Gestational age (weeks)				
	Mean ±SD	6.92 ± 2.39	5.7 ± 2.38	0.01
	≤ 4	21 (10.66%)	14 (28.00 %)	0.01
	5-8	135 (68.53%)	30 (60.00%)	
	9-12	35 (17.72%)	6 (12.00%)	
	≥ 13	6 (3.05%)	0	
β-hCG(IU/ml)				
	Mean ±SD (range)	1851.29 ± 3429.86 (3.9-20000)	3063.09 ± 3849.67 (21-16800)	0.03
	≤1500	139 (70.56%)	18 (36.00%)	< 0.0001
	1501-5000	40(20.30%)	23 (46.00%)	
	≥ 5000	18 (9.14%)	9 (18.00%)	
Normal pregnancies history				
	None	113(57.36%)	23 (46.00%)	0.28
	1	48(24.47%)	12 (24.00%)	
	2	27 (13.71%)	12 (24.00%)	
	3	9 (4.57%)	3 (6.00%)	
Ectopic pregnancies history				
	Yes	17 (8.63 %)	4 (8.00 %)	0.88
Abortion history				
	None	154 (78.17 %)	37 (74.00%)	0.79
	1	32 (16.24%)	9 (18.00 %)	
	2	10 (5.08 %)	4 (8.00%)	
	3	1 (0.51%)	0	
PID history				
	Yes	3 (1.52%)	0	0.38
IUD use				
	Yes	9 (4.57%)	2 (4.00%)	0.86

Results are expressed as mean±SD and percentage (%). Comparison of maternal, obstetric history and maternal serum β-hCG concentrations among two subgroups was performed by; 1) Student’s t-test and Mann-Whitney-Wilcoxon test and 2) Pearson’s chi-square and Fisher’s exact test.

IUD: intrauterine contraceptive device

PID: pelvic inflammatory disease.

**Table II:** Multiple regression analysis with tubal rupture as the independent variables

**Step ** a	**B**	**SE**	**p-value**	**Odds Ratio**	**95% CI**	
					**Lower**	**Upper**
β-hCG > 1750 vs. < 1750 (IU/ml)	1.46	0.34	0.000	4.314	2.221	8.379
Age >35 vs. <35	1.02	0.39	0.009	2.779	1.293	5.972

Dependent Variable: Tubal rupture

Note: β: standard regression coefficient

SEM: standard error of the mean

CI: confidence interval

a . Variable(s) entered on step 1: Gravida, Parity, Abortion, EP history, PID history, IUD history, β-hCG –cutoff, Gestational Age Group, Age Group

## Discussion

In the noninvasive management of a tubal pregnancy, the clinician should focus on selecting those patients in whom the risk of tubal rupture was higher. The occurrence of tubal rupture in EP ranges from 18.0% to 64.5% as reported in previous large population-based studies. The high rupture rates may be caused by lack of diagnostic tools such as transvaginal ultrasound and β-hCG measurements or to delayed diagnosis (10, 11). The incidence of tubal rupture was 20.2% in the present study. We can consider that this rupture rate may be secondary to delayed diagnosis due to no clinical signs or symptoms prior to rupture.

In our study, the mean age of women with ruptured was older than women with unruptured tubal ectopic pregnancy (p<0.0001). Therefore, middle-age is important risk factors for tubal rupture. In Berlingieri *et al* study, prevalence of women with rupture EP was 29.5% and the mean age of these patients was more significantly compared to unruptured EP (12). In contrast to these findings, Goksedef *et al* and Roussos *et al* reported that the mean age of women with ruptured was similar to women with unruptured tubal ectopic pregnancy (1, 13). It seems that older women have more risk to ruptured EP, because unwanted pregnancy and irregular menses are more prevalent in them. Therefore they refer to us with delay. The present study revealed that higher β-hCG levels at the time of admission were important risk factors for tubal rupture. However, no significant associations between parity, gravidity, the number of previous normal pregnancies, past history of PID, previous EP, abortion, IUD use and risk of tubal rupture were found.

We have shown that patients with β-hCG levels >1750 IU/ml were considerably more likely to undergo rupture. Mol *et al* likely suggested that the probability of predicting risk of tubal rupture or active bleeding is over 10% when gestational age is >10 weeks and serum β-hCG levels >8500 IU/m. However, the serum β-hCG cut-off level (>1300 IU/ml) in Mol *et al* study was lower than our study (14). In another study, Downey *et al* showed that a serum β-hCG level of 1500 IU/l is associated with a higher rate of tubal rupture than a β-hCG level of <1.500 IU/l (15). Goksedef *et al* also showed patients with ruptured EP are more frequently to have β-hCG levels of 1501-5000 IU/ml and >5000 IU/ml compared with 0-1500 IU/ml (44.3%, 53.3% and 11.3%, respectively) (1). Therefore, these findings were shown that the serum beta-hCG cut-off level of our study is approximately higher than other studies. 

Nonetheless, there have been sporadic reports of women with ruptured ectopic pregnancies associated with low and declining levels of β-hCG (16). Galstyan *et al* showed that there is no safe β-hCG titer for ruptured tubal ectopic pregnancy and the range of serum β-hCG level was broad for both ruptured and unruptured groups (17). Trophoblast degeneration with cessation of hormone production, an extremely small mass of chorionic villi (producing little if any hormone) or defective β-hCG biosynthesis have been supposed as theoretical mechanism to explain unexpectedly low or absent β-hCG in these patients (16). 

Regarding to relation of gestational age and tubal rupture, significantly higher mean gestational age has previously been demonstrated in ruptured compared with unruptured EP (10, 11). Goksedef *et al *showed women with gestational age 6-8 weeks are 3.6 times, and with gestational age >8 weeks are 46.6 times, more likely to experience a tubal rupture than those with gestational age <6 weeks (1). However, we have shown a negative correlation between gestational age and tubal rupture. It seems that this finding is likely due to unlikelihood number of patients with rupture compared to unruptured tubal ectopic pregnancies in our study.

Furthermore, we found no significant associations between ruptured and parity, gravidity, PID history, previous EP and IUD use. However, in Berlingieri *et al* study in ruptured group was significantly higher than unruptured group (p=0.006) (12). This finding is against Goksedef *et al* study that parity was similar in study groups (1). Furthermore, a strong relation has been shown between past history of EP and rupture risk in literature (18, 19). It seems that our patients with history of EP had more information about clinical feature, so they refer to us before rupture. There are no association between IUD use and PID history and risk of rupture EP in most literature (1, 11, 12).

## Conclusion

In conclusion, based on our results, β-hCG concentration is the best predictive factor for rupture in tubal ectopic pregnancies, which may contribute to the choice of treatment approaches for women who desire future pregnancy. It is recommended to accomplish more studies to determine the level of serum β-hCG on which risk of rupture is more significantly.

## Note

***This article extracted from M.D. thesis. (Ali*** ***Akbar Alipoor)***
